# Artificial intelligence in the diagnosis and management of gynecologic cancer

**DOI:** 10.1002/ijgo.70094

**Published:** 2025-04-25

**Authors:** Chaiyawut Paiboonborirak, Nadeem R. Abu‐Rustum, Sarikapan Wilailak

**Affiliations:** ^1^ Department of Obstetrics and Gynecology Bangkok Metropolitan Administration General Hospital (Klang Hospital) Bangkok Thailand; ^2^ Gynecology Service, Department of Surgery Memorial Sloan Kettering Cancer Center New York New York USA; ^3^ Department of OB/GYN Weill Cornell Medical College New York New York USA; ^4^ Department of Obstetrics and Gynecology, Faculty of Medicine Ramathibodi Hospital Mahidol University Bangkok Thailand

**Keywords:** artificial intelligence, cervical cancer, diagnostic imaging, early detection, endometrial cancer, gynecologic oncology, ovarian cancer, personalized medicine, robotic surgery

## Abstract

Gynecologic cancers affect over 1.2 million women globally each year. Early diagnosis and effective treatment are essential for improving patient outcomes, yet traditional diagnostic methods often encounter limitations, particularly in low‐resource settings. Artificial intelligence (AI) has emerged as a transformative tool that enhances accuracy and efficiency across various aspects of gynecologic oncology, including screening, diagnosis, and treatment. This review examines the current applications of AI in gynecologic cancer care, focusing on areas such as early detection, imaging, personalized treatment planning, and patient monitoring. Based on an analysis of 75 peer‐reviewed articles published between 2017 and 2024, we highlight AI's contributions to cervical, ovarian, and endometrial cancer management. AI has notably improved early detection, achieving up to 95% accuracy in cervical cancer screening through AI‐enhanced Pap smear analysis and colposcopy. For ovarian and endometrial cancers, AI‐driven imaging and biomarker detection have enabled more personalized treatment approaches. In addition, AI tools have enhanced precision in robotic‐assisted surgery and radiotherapy, and AI‐based histopathology has reduced diagnostic variability. Despite these advancements, challenges such as data privacy, bias, and the need for human oversight must be addressed. The successful integration of AI into clinical practice will require careful consideration of ethical issues and a balanced approach that incorporates human expertise. Overall, AI presents significant potential to improve outcomes in gynecologic oncology, particularly in bridging healthcare gaps in resource‐limited settings.

## 1 INTRODUCTION

Gynecologic cancers, including cervical, ovarian, and endometrial cancers, remain a significant global health challenge. In 2020, cervical cancer affected 604 127 women globally, resulting in 341 831 deaths, with an age‐standardized incidence of 13.3 cases per 100 000 women and a mortality rate of 7.2 per 100 000.^1^ Endometrial cancer accounts for 2.1% of new cancer cases and 1.0% of cancer deaths, while ovarian cancer represents 1.6% of new cases and 2.1% of deaths worldwide.^2^ These statistics underscore the need for improved prevention, early detection, and treatment strategies.

Early diagnosis is vital for better outcomes. For instance, early‐stage cervical cancer can often be cured with surgery and radiotherapy. Similarly, early detection of ovarian and endometrial cancers allows for more effective management with a combination of surgery, chemotherapy, and radiotherapy. However, traditional diagnostic methods, such as Pap smears and biopsies, often face challenges in sensitivity and accessibility, especially in low‐resource settings.^2^, ^3^

Artificial intelligence (AI) has emerged as a valuable tool in gynecologic oncology that enhances screening by identifying high‐risk individuals and supports clinicians with real‐time decision‐making. Integrating AI into clinical practice is critical for improving global gynecologic cancer care.^4^

## 2 OVERVIEW OF AI AND ITS POTENTIAL IN HEALTH CARE

### 2.1 A snapshot of AI's development

**FIGURE 1:**
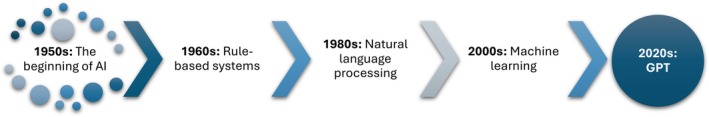
A snapshot of AI's development. AI, artificial intelligence; GPT, generative pre‐trained transformer.

AI has significantly evolved since Alan Turing and John von Neumann laid its theoretical foundations in the 1950s (Figure 1). Turing's introduction of the “Turing Test” set a benchmark for assessing machine intelligence. The formal birth of AI as a field occurred in 1956, when John McCarthy coined the term “artificial intelligence” during the Dartmouth Conference.^5^, ^6^ The 1960s introduced rule‐based and expert systems that encoded human knowledge through logical reasoning. By the 1980s, natural language processing emerged, enabling machines to understand and generate human language. The 2000s witnessed a revolution with machine learning (ML) and big data, which enhanced AI's ability to process vast amounts of information. The 2020s marked a breakthrough with generative pre‐trained transformer‐3 (GPT‐3), a powerful model capable of generating highly coherent and contextually relevant text, which paved the way for advanced language models like GPT‐4.^7^

### 2.2 Generative AI explained

AI encompasses technologies that enable machines to perform tasks that require human‐like intelligence, such as learning and problem‐solving.^6^ It includes ML, which involves teaching algorithms to recognize patterns and make decisions. Deep learning (DL), a branch of ML, uses layered neural networks for complex tasks like image and speech recognition.^8^ Generative AI, which is a subset of AI, focuses on creating new content, such as text and images, using models like generative adversarial networks and variational autoencoders.^7^ These technologies continue to advance, enhancing their capabilities and applications.

### 2.3 Comparative trends in AI publications: Health care versus gynecologic oncology

In recent years, there has been a rapid increase in the number of publications about AI use in health care, highlighting the expanding role of the technology in medical fields. A PubMed search in 2023 identified 23 306 articles on this topic, with significant attention given to imaging specialties such as radiology and ophthalmology.^9^ AI research in non‐gynecologic cancers, including colorectal and breast cancer, has more publications and larger datasets compared to gynecologic cancers.^10^, ^11^, ^12^ Between 2010 and 2018, the number of AI publications in gynecologic oncology peaked at 89 in 2013, before declining to 53 by 2018. In contrast, non‐gynecologic oncology publications reached 1884 in 2018, indicating a substantial focus on other cancer types.^13^ Ovarian cancer exhibited the highest average annual increase in publications at 15.4%, while cervical and endometrial cancers had increases of 13.02% and 12.51%, respectively.^14^ Despite the lower number of publications, the development of AI‐driven diagnostic tools and personalized medicine in oncology continues to grow.

### 2.4 AI in transforming clinical practice

Automated AI systems are transforming clinical workflows by generating accurate, standardized reports and integrating them seamlessly into electronic health records (EHRs). AI‐driven automation is revolutionizing clinical reporting, pathology analysis, and multidisciplinary decision‐making in gynecologic oncology. The integration of ML and natural language processing (NLP) enhances workflow efficiency, reduces clinician workload, and improves decision support.^15^

A major advancement in EHR integration is the “Blue‐button” tool, a prospective AI‐based system developed by the American Cancer Society Cancer Action Network. This tool automatically identifies eligible patients for clinical trials by integrating with existing trial‐matching platforms like CareBox, reducing manual screening efforts. A 12‐month study is currently evaluating whether this system increases trial participation and diversity.^15^ Similarly, Epic Systems Corporation—an American privately held healthcare software company based in Verona, Wisconsin—has incorporated generative AI to assist in billing, patient communication, and structured clinical documentation, streamlining administrative processes while improving data accuracy.^15^

By enabling real‐time AI‐assisted reporting, automated pathology assessments, and seamless EHR integration, AI is reshaping oncologic workflows, ensuring faster decision‐making, better clinical coordination, and improved patient outcomes. As AI technology advances, its adoption in clinical reporting and automation will be instrumental in optimizing cancer care and healthcare resource utilization.^16^

## 3 ROLE OF AI IN SCREENING AND EARLY DETECTION

AI is rapidly transforming the landscape of gynecologic oncology, particularly in the early detection of cervical, endometrial, and ovarian cancers. AI‐driven algorithms are now being integrated into clinical practice, offering unparalleled diagnostic precision that surpasses traditional methods. Recent clinical trials have shown the potential of AI in enhancing patient recruitment, monitoring treatment efficacy, and improving outcomes in gynecologic oncology. AI‐driven models are increasingly utilized to optimize trial screening, improve enrollment efficiency, and reduce bias. A recent study analyzing gynecologic oncology clinical trial sites found that 13% of sites have integrated AI into screening processes, while 60% systematically track screening data to assess feasibility and improve patient matching. AI‐powered EHR integration further facilitates automated eligibility screening, reducing manual workload and enrollment delays .^15^

### 3.1 AI in cervical cancer screening

Cervical cancer screening is crucial for early detection, and the integration of AI has significantly enhanced this process. AI‐driven tools, such as automated Pap smear analysis and advanced colposcopy, have notably improved diagnostic precision, reduced human error, and increased consistency. Recent advancements in AI have further refined the ability to distinguish between cancerous and normal Pap smears, with reported accuracies in the range of 70%–100%.^17^

AI has also shown substantial potential in detecting HPV types and associated molecular markers, which are critical in diagnosing cervical lesions. Because different HPV types are linked to specific lesions, accurately identifying these types can enhance the classification and management of HPV‐infected women. For instance, Wong et al.^18^ demonstrated that incorporating additional genotyping of other high‐risk HPVs into outcome classifiers improved the specificity of identifying cervical intraepithelial neoplasia (CIN) 2/3 lesions to 94.32%, providing a more precise decision‐making tool for clinical management.

### 3.2 Automated cervical smear analysis

AI models have significantly advanced cervical cancer detection, delivering accuracy beyond traditional methods. The U‐Net model, specifically designed for automatic segmentation, has shown exceptional performance in noisy Pap smear images. Nazir et al.^19^ reported a Dice coefficient of 0.894 ± 0.022, demonstrating the model's high accuracy in segmenting intermediate squamous cells despite the presence of Poisson noise.^19^ Similarly, the AlexNet model, when combined with region‐of‐interest extraction, achieved a classification accuracy of 95.78% and a specificity of 94.82% in identifying cellular nuclei in Pap smear images.^20^

In large‐scale cervical cancer screening, AI models have greatly improved accuracy and efficiency. In a study involving 703 103 women, AI‐assisted cytology systems demonstrated a concordance rate of 94.7% with manual cytology and increased sensitivity for detecting CIN2 positivity by 5.8%, with only a slight reduction in specificity.^21^ In addition, Hou et al.^22^ found that AI systems achieved detection rates of 92.6% for CIN2 positivity and 96.1% for CIN3 positivity, significantly outperforming manual cytology in sensitivity and efficiency. These advancements highlight the transformative potential of AI in cervical cancer screening, offering faster, more accurate, and more reliable diagnostics, and ultimately improving patient outcomes.

### 3.3 Enhanced accuracy in colposcopy

Recent advancements in AI have significantly enhanced the accuracy of colposcopy by improving image analysis and lesion detection. AI‐driven systems offer more consistent interpretations, which is especially beneficial in low‐resource settings. These systems facilitate the detection of high‐grade cervical lesions and guide biopsy procedures, improving diagnostic precision and patient outcomes. In low‐ and middle‐income countries, where trained colposcopists are limited, AI‐guided colposcopy presents an effective solution to improve cervical cancer screening.^23^

For improving image analysis, the application of convolutional neural networks (CNNs) in colposcopy has demonstrated superior performance, with one study reporting an AI‐assisted accuracy of 94.1%, sensitivity of 95.6%, and specificity of 83.3% in detecting high‐grade squamous intraepithelial lesions.^24^ In addition, the C‐RCNN algorithm by Yue et al.^25^ demonstrated exceptional performance in diagnosing CIN grades, achieving 96.13% accuracy, 98.22% specificity, and 95.09% sensitivity. These advancements underscore AI's transformative potential in cervical cancer screening, improving both accuracy and efficiency.

To further improve diagnostic accuracy and reduce overtreatment, another study developed a binary decision model to determine the need for biopsy in cervical lesions. The authors defined “need to biopsy” as lesions that were “not normal,” including CIN+ and low‐grade squamous intraepithelial–positive lesions. The best‐performing RESNET‐152 model showed a mean area under the receiver operating characteristic curve (AUC) of 0.947, with a sensitivity of 85.2% and a specificity of 88.2%, effectively aiding inexperienced clinicians in deciding whether to perform a cervical biopsy or refer the patient to a specialist.^26^

### 3.4 AI in ovarian cancer detection

AI has become a valuable tool in improving ovarian cancer detection through ML and DL. AI models outperform traditional methods in identifying ovarian malignancies while also aiding in predictive modeling and biomarker identification. These advancements enhance early diagnosis and personalized care for patients.^27^

### 3.5 Image‐based detection using AI

AI has significantly improved the accuracy of ovarian cancer diagnosis across different imaging modalities, particularly ultrasound, magnetic resonance imaging (MRI), and computed tomography (CT). Ultrasound‐based AI models have shown remarkable diagnostic performance, with a pooled AUC of 0.94 (95% confidence interval [CI] 0.88–1.00), making them especially effective for early detection.^28^ MRI‐ and CT‐based models also perform well, with AUCs of 0.82 (MRI: 95% CI 0.71–0.93; CT: 95% CI 0.78–0.86), but standardization is needed due to high heterogeneity across studies (*I* ^2^ values >90%).^28^

A systematic review and meta‐analysis of 14 studies by Mitchell et al.,^27^ analyzing 15 358 ultrasound images, confirmed AI's strong diagnostic accuracy in detecting ovarian cancer, reporting an overall sensitivity of 81% and specificity of 92%. This highlights AI's potential in improving diagnostic consistency and aiding in early detection, especially in ultrasound‐based examinations. In addition, a more comprehensive meta‐analysis by Xu et al.^29^ involving 34 studies revealed that AI models achieved a pooled sensitivity of 88% and specificity of 85%, with an impressive overall AUC of 0.93, demonstrating AI's ability to outperform conventional diagnostic methods. These findings underscore AI's growing role in enhancing diagnostic precision, reducing human error, and offering a non‐invasive, efficient approach to early ovarian cancer detection.

Despite the variation in study designs, AI models—particularly those using ultrasound—mark a major advancement in diagnosing ovarian cancer. Further research is necessary to standardize AI techniques and confirm their effectiveness across different populations. Integrating AI into clinical practice holds great potential to support earlier diagnosis, improve treatment outcomes, and ultimately enhance the quality of patient care.^27^, ^28^, ^29^

### 3.6 The role of AI in enhancing ovarian cancer biomarker discovery and diagnosis

Ovarian cancer is a highly lethal gynecological malignancy, often diagnosed at advanced stages due to the absence of effective early detection methods.^30^, ^31^ Although biomarkers such as CA‐125 are commonly used, they exhibit limited sensitivity (50%–62%) and specificity (73%–77%) for early‐stage detection, resulting in frequent false negatives and missed diagnoses.^31^, ^32^ These limitations significantly impact the ability to detect ovarian cancer early, which is crucial for improving survival rates. Despite these challenges, there remains a significant gap in comprehensive data on ovarian cancer biomarker discovery. AI‐driven approaches are increasingly being explored to address this gap by analyzing complex datasets to enhance biomarker discovery. ML models, such as support vector machines and random forests, can integrate genomic and proteomic data to improve diagnostic accuracy. For example, Kawakami et al.^30^ demonstrated that random forest classifiers could differentiate ovarian epithelial cancer (OEC) from benign tumors with an accuracy of 92.4% (AUC = 0.968) by analyzing 32 biomarkers from peripheral blood tests, although these models showed lower performance in predicting cancer stages and histologic subtypes.

However, there is currently no specific workflow established for the direct application of AI in enhancing ovarian cancer biomarker discovery and diagnosis. Nonetheless, recent advancements in liquid biopsy techniques have demonstrated significant improvements in diagnostic accuracy. By utilizing circulating tumor DNA (ctDNA), recent studies have achieved a detection sensitivity of up to 93.8%, far surpassing the capabilities of traditional markers such as CA‐125.^32^ This non‐invasive approach holds great promise for earlier diagnosis, personalized treatment strategies, and effective monitoring of disease progression.^31^, ^32^

### 3.7 AI in endometrial cancer screening

Endometrial cancer is the most common gynecologic malignancy in developed countries, with rising incidence due to increasing obesity and aging populations. Early detection and risk stratification are crucial, as early‐stage cancers have high survival rates compared to advanced stages, which have poorer outcomes. Recent advancements in AI show promise in enhancing endometrial cancer screening, particularly in risk prediction and early intervention.

### 3.8 The role of AI in risk prediction

Traditional models for endometrial cancer risk, focusing on age, body mass index (BMI, calculated as weight in kilograms divided by the square of height in meters), and genetics, often lack sensitivity. A 2021 meta‐analysis identified obesity (BMI > 30) as the most significant risk factor, with a relative risk of 2.87 for developing endometrial cancer. The study also used a neural network model to predict cancer diagnoses with an accuracy over 98%, greatly improving traditional methods.^33^ In 2023, an ML model incorporating immune landscape data and traditional prognostic factors achieved an accuracy of 69%, significantly enhancing the prediction of recurrence and survival outcomes by including tumor mutational burden and immune cell populations.^34^

### 3.9 AI‐driven early intervention strategies

AI has shown great potential in diagnosing endometrial cancer, especially in postmenopausal women with symptoms like vaginal bleeding and endometrial thickness (≥5 mm). A case–control study comparing diagnostic models found artificial neural networks to be the most accurate, with a sensitivity of 86.8%, specificity of 83.3%, and overall accuracy of 85.4%, outperforming other methods.^35^ These findings highlight AI's role as a non‐invasive screening tool, supporting clinicians in primary care decisions by combining genetic, molecular, and clinical data for personalized prevention strategies.

### 3.10 Histopathology‐based endometrial cancer tailored outcome risk model for recurrence prediction

The histopathology‐based endometrial cancer tailored outcome risk (HECTOR) model, developed using DL on data from 2072 patients, achieved C‐indices up to 0.828. It effectively stratified patients into low‐, intermediate‐, and high‐risk groups for recurrence, with 10‐year distant recurrence‐free probabilities in the range of 58.1%–97.0%. HECTOR also identified patients who would benefit from adjuvant chemotherapy, surpassing current methods in guiding personalized treatment.^36^

AI is transforming endometrial cancer screening, improving precision in risk stratification and guiding early interventions. As AI technologies evolve, their integration into clinical practice will be essential for advancing early detection and improving outcomes for women at risk of endometrial cancer.

## 4 AI IN DIAGNOSTIC IMAGING

### 4.1 Radiomic analysis and tumor characterization

Radiomics is an innovative tool in gynecologic oncology that extracts quantitative data from medical images to enhance diagnosis, prognosis, and treatment planning, especially in cervical and ovarian cancers.

In cervical cancer, radiomics features from MRI and ultrasound can accurately predict lymph node metastasis and VEGF expression. Combining radiomics with clinical data further improves predictions of lymphovascular space involvement and survival outcomes, providing a more comprehensive tumor profile.^37^, ^38^

For ovarian cancer, CT‐based radiomics models have demonstrated correlations with survival metrics, including overall survival and progression‐free survival, though larger studies are needed to confirm these findings for clinical implementation.^39^ Radiomics provides a non‐invasive, three‐dimensional (3D) view of tumors, which can help in reducing reliance on biopsies and supporting personalized treatment plans.^40^ Advanced imaging techniques, such as 68Ga‐FAPI‐PET/CT, have shown enhanced tumor‐to‐background contrast, particularly for metastatic lesions in bone, liver, and lymph nodes, compared to conventional imaging methods.^41^ This increase in contrast supports improved tumor characterization and reinforces radiomics' potential to refine gynecologic cancer care through a more tailored, patient‐centered approach.

### 4.2 Automated tumor segmentation: AI‐enhanced MRI and CT imaging

In gynecologic oncology, AI‐driven tools, particularly those utilizing DL models, allow for the automatic segmentation of tumors in cervical and endometrial cancers with a precision comparable to that of expert radiologists. This not only enhances diagnostic accuracy but also streamlines workflows, enabling timely and reliable volumetric measurements that are essential for personalized treatment planning.^42^, ^43^

The adoption of AI‐based segmentation models, such as CNNs, has demonstrated impressive results in clinical settings, achieving segmentation accuracy exceeding 90% in cervical and endometrial cancers.^42^, ^43^ Furthermore, AI applications extend to more complex imaging contexts, such as positron emission tomography (PET)/CT scans in lymphoma, in which they enhance diagnostic confidence by up to 15%.^44^, ^45^

AI's influence extends further to MRI‐guided gynecologic brachytherapy, where automated segmentation of brachytherapy catheters is proving transformative. According to Zaffino et al.,^46^ the application of a 3D CNN model for catheter segmentation achieved a mean Dice score of 0.60 and reduced false positives to 6.7% and false negatives to 1.5%. This method allows for accurate and efficient identification of closely spaced catheters, which is crucial given that the number of catheters per patient can be range in the range of 10–35. By automating catheter segmentation, this AI tool significantly optimizes radiation delivery, reducing treatment planning time and enhancing overall patient outcomes. In addition, the implementation of this model in open‐source platforms like 3D Slicer facilitates broader clinical adoption and continued research in MRI‐guided brachytherapy.^46^

Radiomics, powered by AI, has demonstrated high accuracy in identifying tumor characteristics, predicting treatment response, and stratifying patients by risk. AI‐enhanced radiomics models applied to contrast‐enhanced CT imaging have achieved an AUC of 0.91–0.96 in classifying ovarian cancer subtypes, while MRI‐based AI models have demonstrated an AUC of up to 1.00 in distinguishing borderline ovarian tumors from malignant lesions.^47^ Beyond diagnostics, AI‐integrated radiogenomics has enhanced predictive modeling for chemotherapy response and overall survival, offering valuable decision support in personalized treatment planning.^47^

In summary, AI‐enhanced tumor segmentation is streamlining diagnostic and therapeutic processes in gynecologic oncology, leading to more accurate diagnoses, better treatment personalization, and ultimately improved patient outcomes. As AI technology continues to advance, its role in clinical practice is poised to expand significantly.^45^, ^46^, ^47^, ^48^, ^49^, ^50^

## 5 AI IN HISTOPATHOLOGY: DIGITAL PATHOLOGY AND AI IN GYNECOLOGIC CANCERS

Digital pathology and AI have significantly advanced cancer diagnostics, particularly in gynecologic cancers. AI has transformed two key areas: automated tissue analysis and genetic mutation prediction.

### 5.1 Automated tissue analysis

Digital pathology has revolutionized traditional microscope‐based evaluations by digitizing whole‐slide images, making analysis more efficient and scalable. A key AI application in this area is automated tissue analysis, which utilizes DL algorithms like CNNs to classify and quantify histopathological features in tissue samples. In gynecologic cancers, AI has shown remarkable accuracy in automated analysis. For example, AI models for classifying ovarian cancer subtypes have achieved up to 90% accuracy, significantly reducing the variability and subjectivity of manual assessments. One DL model even reached an impressive 97% accuracy in classifying ovarian cancer subtypes.^1^ In a study by Wu et al.,^48^ the authors used AI to analyze hematoxylin and eosin (H&E)‐stained tissue samples from 85 cases of ovarian cancer, resulting in an accuracy of 0.78, verified by pathologists. In addition, multidisciplinary tumor boards benefit from AI‐driven language models, such as ChatGPT, which help standardize treatment recommendations and align decisions with clinical guidelines.^51^

AI's integration into clinical workflows can assist pathologists by offering second‐opinion diagnostics, particularly in settings with limited resources. Automated analysis reduces time, minimizes errors, and allows pathologists to focus on more complex cases. Moreover, AI‐based tools enable quantitative biomarker analysis, supporting personalized treatment strategies and improving patient outcomes.^46^, ^49^

### 5.2 AI for genetic mutation prediction

The role of AI extends beyond visual analysis to include the prediction of genetic mutations from histopathological images. This approach has significant potential, as identifying genetic alterations such as *TP53* or *BRCA* mutations can guide treatment decisions in gynecologic cancers. Traditionally, molecular testing has been the gold standard for identifying these mutations, but AI has demonstrated the ability to predict genetic mutations directly from digitized pathology slides, which could reduce the need for expensive molecular tests.^50^

For instance, a study using AI to predict *BRCA1* and *BRCA2* gene mutations in ovarian cancer achieved an AUC of 0.95 and 0.91, respectively, supporting the stratification of patients for personalized treatment.^52^ However, a review of 24 studies found that the overall average AUC for gene mutation predictions was 0.64, underscoring the need for larger, more diverse datasets to improve AI's accuracy in clinical practice.^53^

Despite AI's potential to improve diagnostics and treatment strategies, challenges remain, including algorithm transparency and data quality concerns. Further research and multicenter trials are necessary to refine AI tools for widespread clinical use.

## 6 AI IN PERSONALIZED TREATMENT PLANNING

### 6.1 AI in surgical planning

#### Robotic‐assisted surgery

In gynecologic cancer surgery, AI‐enhanced robotic systems have revolutionized surgical precision and decision‐making. The da Vinci Surgical System, a leading platform, utilizes AI to improve motion control, reduce human error, and enhance precision in procedures such as hysterectomies and pelvic lymph node dissections. These AI‐driven systems significantly improve surgical safety and accuracy, reducing postoperative complications compared to traditional open surgery.^54^, ^55^

During surgery, AI algorithms process real‐time data to predict tissue behavior, dynamically adjusting robotic movements. For instance, the Smart Tissue Autonomous Robot achieved an impressive 87% success rate in autonomous soft tissue surgeries, outperforming human surgeons in accuracy during trials.^56^ This level of precision is crucial in ensuring clear surgical margins, which helps reduce cancer recurrence—a key concern in gynecologic oncology.^57^, ^58^

Finally, in surgical oncology, AI‐assisted Optical Coherence Tomography (OCT) enables real‐time intraoperative tissue assessment with diagnostic sensitivities in the range of 66.7%–94%, specificity of 64%–100%, and an accuracy of 73%–96%.^59^ AI‐driven Full‐Field OCT (FF‐OCT) and Dynamic Cell Imaging (DCI) have been integrated into oncologic surgery, providing rapid, high‐resolution tumor margin assessment in under 5 minutes, improving surgical precision and minimizing unnecessary tissue resection.^59^

#### Preoperative planning and navigation

AI plays an essential role in preoperative planning, leveraging imaging technologies like CT, MRI, and PET/CT to generate highly detailed, 3D anatomical models. These models enable surgeons to create personalized surgical plans, minimizing complications and improving outcomes. In gynecologic cancer, in which tumor location and surrounding anatomy vary significantly, AI's ability to tailor surgical plans is invaluable. Studies have shown that AI‐based planning can reduce intraoperative complications and improve surgical precision, leading to better postoperative recovery and outcomes.^55^, ^60^

AI‐guided navigation systems provide real‐time feedback during surgery by incorporating preoperative imaging data and tracking tissue interactions and instrument positioning. These systems significantly enhance surgical precision, particularly in oncologic procedures where accurate tumor resection is critical. In gynecologic surgeries, where precise removal of tumors is essential for optimal patient outcomes, AI‐driven navigation can optimize surgical decision‐making and reduce the likelihood of complications.^55^, ^57^

### 6.2 AI in radiotherapy

AI has transformed radiotherapy in gynecologic cancers by optimizing treatment planning, improving precision, and reducing toxicity. Techniques such as intensity‐modulated radiotherapy and stereotactic body radiotherapy allow for more precise radiation delivery to tumors while sparing healthy tissues. Intensity‐modulated radiotherapy, for example, modulates radiation beam intensity and angle to deliver high doses to the tumor while minimizing exposure to surrounding organs like the bladder and bowel, which is critical in gynecologic cancers.^61^, ^62^ Stereotactic body radiotherapy delivers high‐dose radiation in fewer sessions, often with sub‐millimeter precision, making it particularly effective for smaller tumors or cases for which surgery is not feasible.^63^ AI‐driven radiotherapy techniques in cervical and endometrial cancers have shown local control rates exceeding 80%, with dose optimization reducing organ‐at‐risk exposure by 20%.^62^ AI's predictive models also enhance adaptive radiotherapy, which modifies treatment plans in real time based on imaging changes during the treatment, further improving local control rates and cutting planning time from day to hours.^64^

Moreover, AI plays a crucial role in optimizing resource utilization in radiotherapy. By integrating predictive models and automated processes, AI reduces planning time and improves overall workflow efficiency, thus minimizing delays in treatment delivery. AI also assists in balancing machine workloads and managing patient data, reducing unnecessary resource use and improving patient outcomes, particularly in busy radiotherapy centers.^65^ Despite these advancements, challenges remain, particularly in reducing side effects. Grade 3–4 gastrointestinal toxicity still occurs in up to 20% of cases during salvage treatments for pelvic recurrences.^63^ Nonetheless, AI continues to advance the field, improving outcomes and reducing complications in radiotherapy for gynecologic cancers.

### 6.3 AI in chemotherapy

AI is also making strides in chemotherapy, enabling more accurate predictions of treatment response, which helps tailor personalized therapies. In ovarian cancer, multi‐gene expression predictors can differentiate between responders and non‐responders, which significantly affects survival outcomes. A study revealed that the median survival for responders was 55.4 months compared to 32.1 months for non‐responders (*P* = 0.014).^66^

AI models, including DL techniques, are being employed to predict chemotherapy responses in complex cancers, such as triple‐negative breast cancer. A deep CNN, trained to analyze whole‐slide images of H&E‐stained tissue sections, has shown impressive predictive performance, especially in early‐stage disease, with an AUC of 0.88.^67^

Similarly, an ML model developed by Mucaki et al.^68^ successfully predicted carboplatin resistance in ovarian cancer. The model identified key gene signatures such as *AKT1*, *TP53*, and *VEGFB*, which showed significant predictive accuracy for both disease recurrence and remission. These insights into chemoresistance mechanisms enable more personalized treatment approaches, helping clinicians select the most effective chemotherapy regimens.

Dynamic biomarkers like CA‐125 kinetics are also being incorporated into AI models. In ovarian cancer, early changes in CA‐125 levels have been closely linked to survival outcomes, with the CA‐125 elimination rate constant proving more predictive than traditional criteria.^69^ By integrating such biomarkers, AI can offer real‐time assessments of chemotherapy efficacy, supporting clinicians in adjusting therapies for optimal outcomes.

As AI models continue to evolve and be validated, their incorporation into clinical practice could revolutionize cancer treatment, offering more personalized and effective therapeutic recommendations based on a comprehensive analysis of gene expression, histopathological imaging, and dynamic biomarkers.

### 6.4 AI in immunotherapy

AI is transforming cancer vaccine development and immunotherapy. AI‐assisted computational models have identified novel immune adjuvants, with some progressing to early‐phase clinical trials. These technologies enhance tumor‐specific antigen prediction, facilitating the design of more effective cancer vaccines. Furthermore, AI‐driven ligand‐based screening methods are being employed to analyze potential immunogenic compounds, optimizing vaccine formulations for enhanced therapeutic efficacy.^16^

These advancements underscore AI's role in clinical trial efficiency, patient‐centered recruitment, and precision oncology, paving the way for more accessible and effective therapeutic strategies in gynecologic cancer care.

## 7 AI IN PATIENT MONITORING

### 7.1 AI in patient monitoring of recurrence in gynecologic cancer

AI and ML models, such as random forest and support vector machines, have demonstrated significant accuracy in predicting cancer recurrence. Kim et al.^70^ developed an ML model utilizing Lynch syndrome‐related markers, achieving an AUC of 0.82 for recurrence prediction in endometrial and ovarian cancers. Similarly, Nithya and Ilango^71^ introduced a classifier model that demonstrated a sensitivity of 85% and specificity of 90% in predicting cervical cancer recurrence.

### 7.2 How AI works in recurrence monitoring

AI systems for monitoring recurrence process large amounts of clinical, genomic, and imaging data to identify patterns that predict cancer recurrence. ML algorithms, trained on historical datasets of patient outcomes and recurrence, analyze new patient data to predict risk. For example:

- Random forest models create multiple decision trees based on different clinical factors such as tumor markers, patient demographics, and treatment history. The model then aggregates the outputs of these trees to generate a highly accurate prediction regarding recurrence.
- Support vector machines classify patient data into recurrence or non‐recurrence categories by finding the optimal hyperplane that separates the two groups, allowing for more precise predictions.

AI integrates diverse data (clinical, pathological, genetic, and imaging) into unified models, offering real‐time, personalized surveillance strategies. It continuously monitors patient data and refines predictions as new information becomes available, enabling early intervention and improved patient outcomes.^70^, ^71^

## 8 AI IN PATIENT EDUCATION

AI‐driven chatbots and decision‐support tools are transforming gynecologic oncology by improving clinical decision‐making and patient education. These AI systems assist in real‐time patient interactions, symptom tracking, and automated report generation.

A feasibility study on ChatGPT‐4 in multidisciplinary tumor board discussions found that its recommendations were correct in 70% of cases based on NCCN guidelines and 60% according to ESGO guidelines.^51^ However, 25% of cases received incorrect recommendations, with significantly lower accuracy for endometrial cancer (20%) compared to ovarian cancer (90%, *P* = 0.005).^51^

Beyond clinical support, AI chatbots enhance patient education and decision‐making. A study comparing ChatGPT and Google's Bard found that while 91.7% of responses were accurate for cervical cancer screening, accuracy dropped to 33.3% for complex treatment‐related queries. Despite their ability to simplify guidelines for patients, these models lack transparency in sourcing and fail to account for regional guideline variations.^72^

To maximize their potential, AI chatbots must improve accuracy in complex cases, integrate source citations, and align with EHRs for personalized recommendations. Expert oversight remains essential to ensure safe, evidence‐based clinical use. With continued advancements, AI chatbots can play a central role in patient‐centered oncology care.

## 9 CHALLENGES AND ETHICAL CONSIDERATIONS IN AI FOR GYNECOLOGIC CANCER

The integration of AI into gynecologic cancer care presents various challenges and ethical concerns, particularly around data privacy, bias, and patient autonomy. Protecting patient data is critical, as AI systems rely on large, sensitive datasets that require anonymization and informed consent to ensure privacy and security.^14^ Bias in AI models is another significant challenge. If the training data lack diversity, the resulting AI algorithms may produce biased outcomes that disproportionately affect underrepresented groups.^13^ In gynecologic oncology, this could exacerbate existing disparities in healthcare access and treatment outcomes. For instance, models trained on predominantly Western datasets may not account for genetic, cultural, or socioeconomic differences in other populations, leading to unequal care. However, this review indicates that approximately one‐third of the studies included data from Asian populations, which may help reduce bias by increasing the diversity of the training datasets. To mitigate this, AI systems must be trained on diverse datasets that reflect a wide range of patient demographics. Regular audits are also necessary to evaluate the fairness of AI systems, ensuring that they provide equitable care across all patient populations.^13^, ^73^, ^74^ In addition, patient autonomy must be respected by ensuring that patients are fully informed about AI's role in their diagnosis and treatment, with explicit consent required for its use.^74^

To fully realize AI's potential in gynecologic oncology, frameworks must be established to mitigate algorithmic bias and promote equitable implementation, particularly in low‐resource settings. AI‐driven technologies must address algorithmic bias, equitable access, and data privacy to ensure ethical deployment. Bias arises when AI models are trained on non‐representative datasets, leading to disparities in diagnostic accuracy and treatment recommendations, particularly for underrepresented populations.^75^

To counteract these challenges, AI systems should be developed using diverse, multi‐ethnic datasets, undergo regular bias audits, and comply with data protection regulations such as GDPR (EU), HIPAA (USA), and Thailand's PDPA. These laws establish data security protocols, patient consent requirements, and restrictions on cross‐border data sharing, ensuring ethical AI integration.^6^ In addition, explainable AI models must be prioritized to enhance clinician trust and patient safety. Implementing these strategies will support equitable, transparent, and ethically responsible AI applications in gynecologic oncology.

## 10 CONCLUSION

Although AI is becoming increasingly efficient and will continue to advance in the future, making processes faster and more accurate, human oversight remains essential. Physicians using AI in patient care must actively monitor and validate AI‐generated outcomes to ensure accuracy in treatment decisions. Moreover, clinicians should continuously update their knowledge of AI advancements to remain up to date with modern technologies while adhering to the core principles of medical professionalism. Maintaining this balance between technological innovation and human expertise is crucial for the safe and ethical use of AI in health care.

## AUTHOR CONTRIBUTIONS

All authors contributed to conception and design of the work, drafting, reviewing and final approval.

## FUNDING INFORMATION

This research was funded in part by the NIH/NCI Cancer Center Support Grant P30 CA008748 to Memorial Sloan Kettering Cancer Center.

## CONFLICT OF INTEREST STATEMENT

NRA‐R reports grants from GRAIL paid to his institution.

## Data Availability

Data openly available in a public repository that issues datasets with DOIs.
